# Criss cross heart: an outflow tract defect?

**DOI:** 10.1186/s13023-025-04096-2

**Published:** 2025-11-24

**Authors:** Ségolène Bernheim, Adrien Borgel, Véronique Pingault, Stanislas Lyonnet, Damien Bonnet, Sigolène M. Meilhac, Lucile Houyel

**Affiliations:** 1 Medico-Surgical Unit of Congenital and Pediatric Cardiology M3C, Necker-Enfants Malades, Assistance Publique des Hôpitaux de Paris, Paris, France; 2 Université Paris Cité, Paris, France; 3 Imagine - Institut Pasteur Unit of Heart Morphogenesis, INSERM UMR1163, Paris, France; 4 Service de Médecine Génomique des Maladies Rares, APHP Centre, Hôpital Necker-Enfants Malades, Paris, France; 5 Laboratory of Genetics of Developmental Anomalies, INSERM UMR 1163, Imagine Institute, Paris, France

**Keywords:** Criss cross heart, Supero-inferior ventricles, Outflow tract, Morphogenesis

## Abstract

**Background:**

Criss-cross heart (CCH) is a rare congenital malformation in which the atrioventricular inflow vectors are approximately perpendicular. CCH is associated with other defects including malposition of the great arteries, supero-inferior ventricles, and ventricular septal defects (VSD). A recent study in a mouse model demonstrates that CCH and associated malformations can be the result of a growth arrest of the outflow tract. In order to confront this hypothesis, we studied 16 cases of criss-cross heart with detailed anatomical description and clinical outcomes.

**Results:**

All patients with criss-cross heart diagnosed in Necker –Enfants Malades Hospital from 1999 to 2022 were included in a retrospective study. Echocardiography, CT scans and anatomical MRIs were reviewed. Segmental analysis according to Van Praagh was SDL in 11 patients, SDD in 4 and SDA in 1. The ventricles were supero-inferior in 12 patients (75%). Ventriculo-arterial connections were always abnormal: double outlet right ventricle in 15/16 with a bilateral conus in 11 and a subaortic conus in 4, transposition of the great arteries in 1. The pulmonary valve was stenotic or atretic in 12 patients (75%). All patients had a VSD opening in the inlet of the right ventricle: inlet only in 8 patients, confluent inlet/outlet in 6, inlet with muscular extension in 2. Fifteen (94%) patients underwent surgery, univentricular repair in 13/16 (81%), biventricular in two.

**Conclusion:**

Criss-cross heart is always associated with a malposition of the great vessels and a VSD, always of the inlet type. Anatomical characteristics are similar to the ones observed in the mouse model for CCH, suggesting similar developmental mechanisms. The malposition of the great vessels might be due to a defective growth of the outflow tract. The constant finding of a VSD of the inlet type is consistent with an abnormal rotation of the atrioventricular canal.

## Introduction

Criss-cross heart (CCH) is a rare congenital cardiac malformation accounting for less than 0.1% of congenital heart defects [1]. CCH is characterized by an anomaly of the atrioventricular connections [2] in which the atrioventricular flow vectors are approximately orthogonal or perpendicular [3] instead of being parallel. The diagnosis is established by echocardiography on an apical four-chamber view when the opening of the two atrioventricular valves cannot be visualized on the same plane. Criss-cross heart is consistently associated with other defects, including supero-inferior ventricles in 30–60% of cases [4, 5], abnormal ventriculo-arterial connections, pulmonary stenosis, straddling atrioventricular valves [2, 6, 7]. An associated ventricular septal defect is almost always present [1]. The literature about CCH, mostly composed of case reports, exhibits considerable heterogeneity in descriptions, lacking an exhaustive account of associated defects, particularly the localization and the anatomic type of the ventricular septal defect.

Since its initial description by Lev et al. in 1961 [8] and the coining of the term “criss-cross” by Anderson et al. in 1974 [9], two main hypotheses have been proposed regarding the developmental origin of CCH. The first one was a twist of the ventricles following septation [9, 10]; the second hypothesis was that the ventricular malposition often associated with CCH would reflect a looping defect [11]. However, a recent study on the first mouse model for CCH [12] indicates that neither of these hypotheses accounts for the developmental mechanism of CCH. In this experimental model, *Greb1l* mutants, CCH was shown to result from a severe growth defect of the outflow tract occurring very early, just after heart looping, which is normal in this model. The shorter length of the heart tube leads to an abnormal rotation of the atrioventricular canal with a spiraling pattern of the endocardial cushions, before the onset of septation, resulting in the orthogonal planes of the two atrioventricular valves characteristic of CCH. It also leads to an abnormal or absent spiraling of the outflow tract cushions, leading to double-outlet right ventricular type of ventriculo-arterial connections. To test this developmental hypothesis in humans, we describe here the detailed cardiac anatomic phenotype of a cohort of 16 consecutive patients with criss-cross heart.

## Methods

All neonates diagnosed with CCH in Necker -Enfants malades Hospital from 1999 to 2022 were included in a retrospective study. Our inclusion criteria were a diagnosis of CCH confirmed by echocardiography in living children that were treated in Necker Hospital.

The echocardiography, CT scans and anatomical MRIs were reanalyzed for the purpose of this study by a referent pediatric cardiologist to validate the diagnosis and the anatomical features.

Criss-cross heart was diagnosed when the two atrioventricular valves could not be seen on the same plane on the apical four-chamber view. We used the segmental analysis developed by Van Praagh [13] to describe the arrangement of the three major segments of the heart. The atrial situs was considered normal (S: solitus), when the morphologically right atrium, defined by the connection of the inferior caval vein, was right-sided; mirror-imaged (I: inversus) when it was left-sided; ambiguous (A), when the morphology of the atria could not be determined with certainty. The ventricles were D-looped (D) when the morphologically right ventricle was right-sided, L-looped (L) when the morphologically right ventricle was left-sided. Chirality was used when the ventricles were supero-inferior: the morphologically right ventricle is considered right-handed (D-looped) when the palm of the right hand can be placed on the ventricular septal surface with the thumb in the inlet and the fingers in the outlet, and left-handed (L-looped) if the palm of the left hand applies with the same criteria on the ventricular septal surface. The position of the great arteries was S (normal) or I (inverted) when the ventriculo-arterial connections were concordant, and D (aortic valve to the right of the pulmonary valve), L (aortic valve to the left of the pulmonary valve), or A (aorta strictly anterior to the pulmonary artery) when the great vessels were malposed or transposed. The location of the ventricular septal defects (VSD) on the right ventricular septal surface was defined according to the IPCCC ICD-11 nomenclature [3, 14]: perimembranous central, inlet, trabecular muscular, or outlet. The conus, defined as a muscular band separating an arterial valve from an atrioventricular valve, was described based on its anatomical position: subaortic, subpulmonary or bilateral [13]. Supero-inferior ventricles were defined by a horizontal position of the ventricular septum. Pulsed and color doppler were used to analyze valvar stenosis and regurgitation. Demographics, type of repair at last follow-up and vital status were collected from patients reports. For genetic analysis, six patients with criss-cross heart underwent whole-genome sequencing, including four trios (proband and both parents) and two duos (proband and mother only). Libraries were prepared using the TruSeq DNA PCR-free kit (Illumina) and sequenced on NovaSeq6000. Sequences were aligned to the reference human genome hg19 and analyzed with an in-house Polyweb bioinformatics pipeline for sequence and structural variants, including 3 structural variant callers [15–17]. Hypothesis of rare monogenic dominant variants in *GREB1L* coding sequence was explored by using stringent filters: variant seen in less than 5 heterozygotes in GnomAD v3.1.2 database [18], in less than 10 projects in the local human genome or exome database, with a medium or strong impact on the protein (truncating, splicing, frameshift and missense variants) and with an allelic ratio > 25%. To detect recessive variants, we adjusted the frequency filters with less than 1000 heterozygotes in GnomAD and less than 50 runs in the local database. Non-coding variants were analysed with NCboost [19]. We notably focussed on the topologically associating domain (TAD) of *GREB1L* (chr18:18,500,000–20,350,000) identified in [20] and *GREB1L* enhancers predicted in the GeneHancer and Vista Enhancer databases. Structural variants were explored by filtering out variants seen in more than 20 projects in the local human genome or exome database. Variant classification followed the recommendations of the American College of Medical Genetics and Genomics (ACMG) and the Association for Molecular Pathology (AMP). This study has been registered in the General Data Protection Register of the APHP under the registration number: 2024 1,022,164,627. This attests to its compliance with the General Data Protection Register.

## Results

### Population

**Fig. 1:**
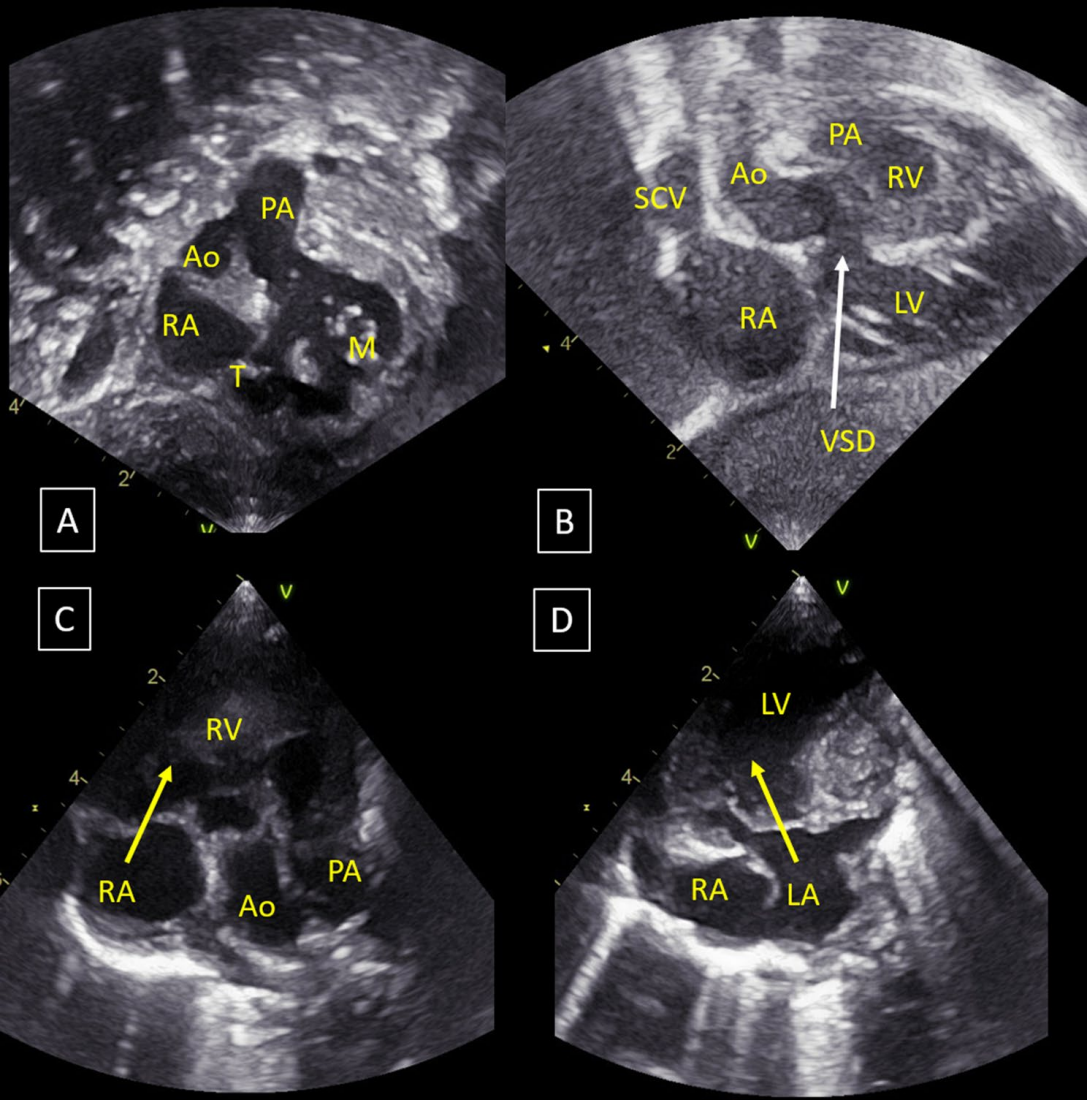
Ultrasound of criss-cross heart with supero-inferior ventricles, double outlet right ventricle (S,D,D), non-committed inlet VSD, hypoplastic right ventricle. (**A**): subcostal view, derived from coronal view. Mitral and tricuspid valves are in orthogonal planes; (**B**): sub costal view, coronal view: horizontal interventricular septum (supero-inferior ventricles); (**C**): 4 chamber view, right atrium-right ventricle connection (yellow arrow); (**D**): 4 chamber view, left atrium-left ventricle connection (yellow arrow); the two yellow arrows are in orthogonal planes. Ao, aorta; LA, left atrium; LV, left ventricle.; M, mitral valve; RA, right atrium; RV, right ventricle; T, tricuspid valve;; PA, pulmonary artery; VSD, ventricular septal defect

Forty-six patients were retrieved from the Necker–Enfants malades Hospital and Imagine Institute congenital heart disease database using DrWarehouse®, a full-text clinical data warehouse for cohort identification and data extraction [21]. Among them, echocardiography report described CCH in 32 patients. Only 21 of these patients had ultrasounds with enough images to permit retrospective analysis. Finally, the diagnosis of CCH was confirmed on the four-chamber view in 16 out of these 21 patients (Fig. 1) and discarded in 5.

Five (31%) patients were female (Table 1). One patient had a history of familial congenital heart defect. No patient had related parents, and no extracardiac anomalies were reported. Six patients underwent genetic testing using whole-genome sequencing. No pathogenic single-nucleotide variants or copy-number variants were identified. We searched specifically for alterations at the *GREB1L* locus, the gene mutated in the criss-cross heart mouse model, including structural and copy number variants, as well as predicted pathogenic variants in exons or non-coding regions of the *GREB1L* TAD. We did not find any genetic anomaly in these patients.

**Table 1:**
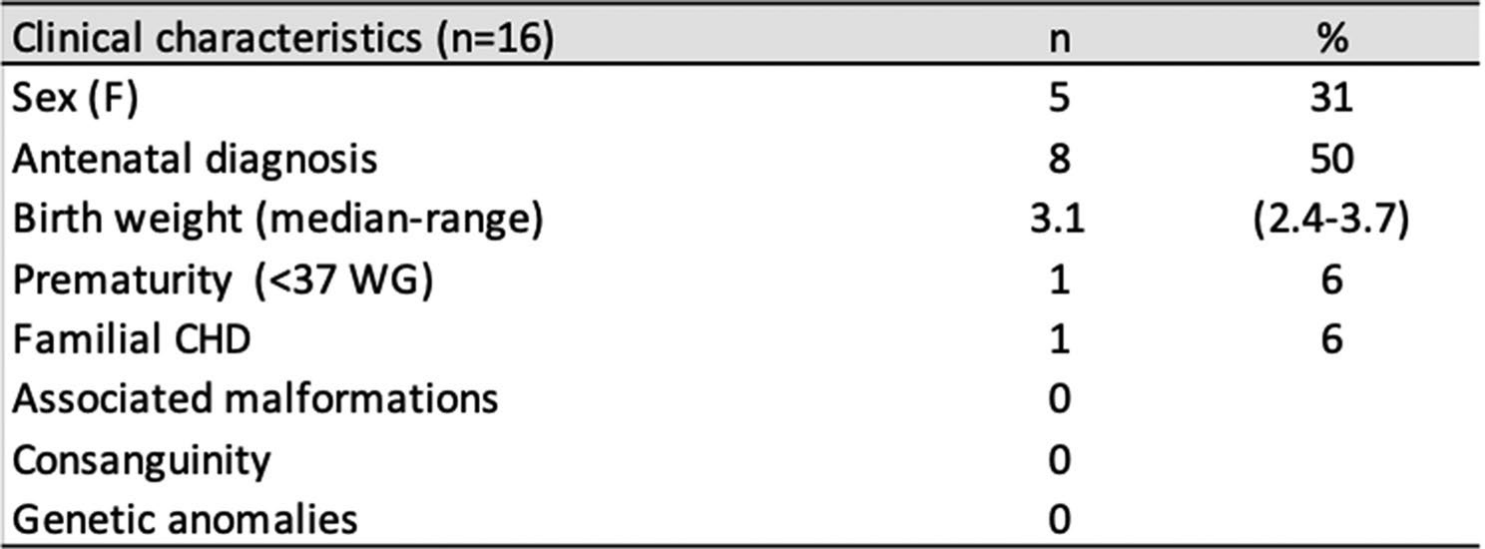
Clinical characteristics of patients with CCH

**Table 2:**
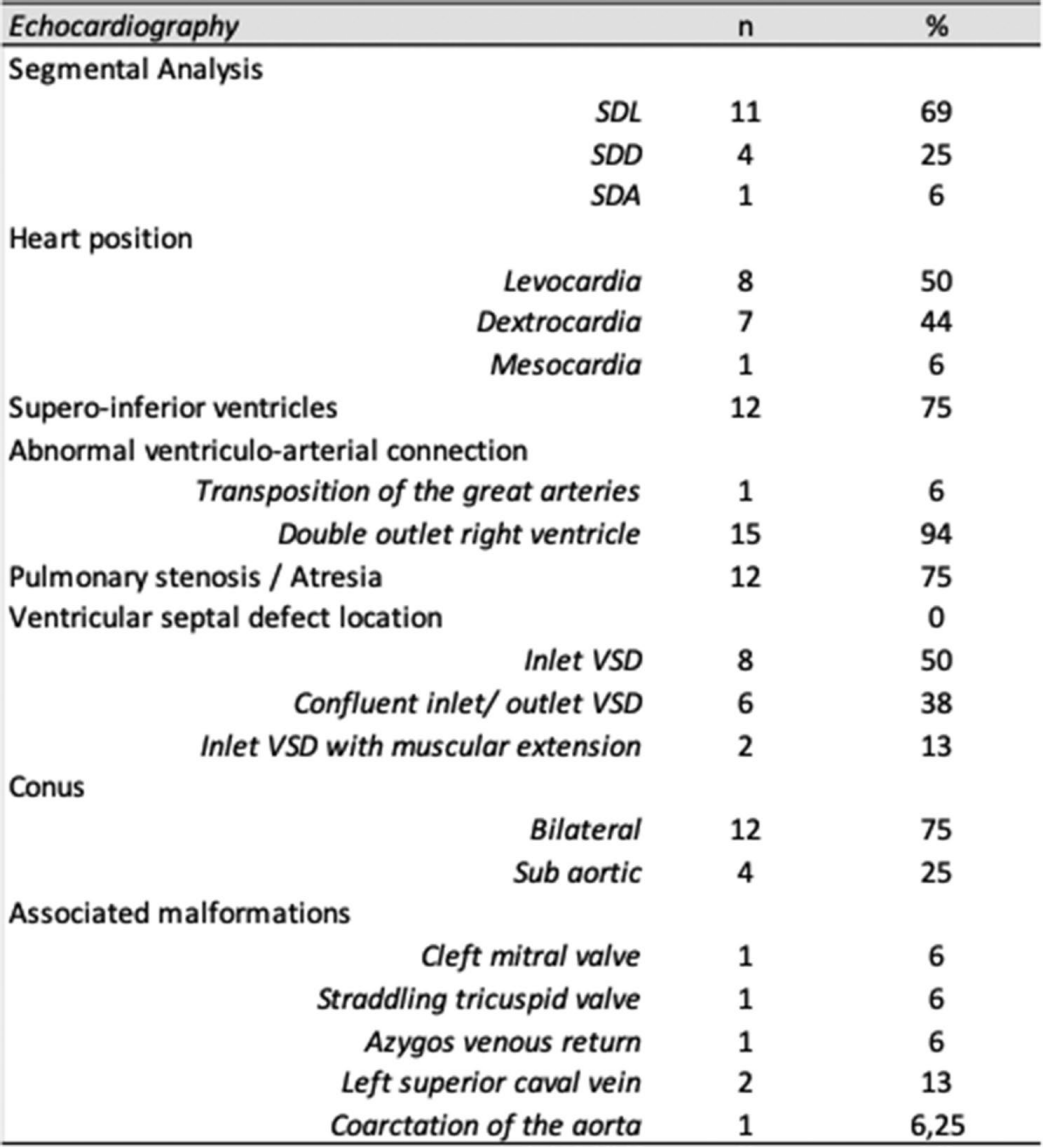
Anatomical characteristics of CCH

**Table 3:**
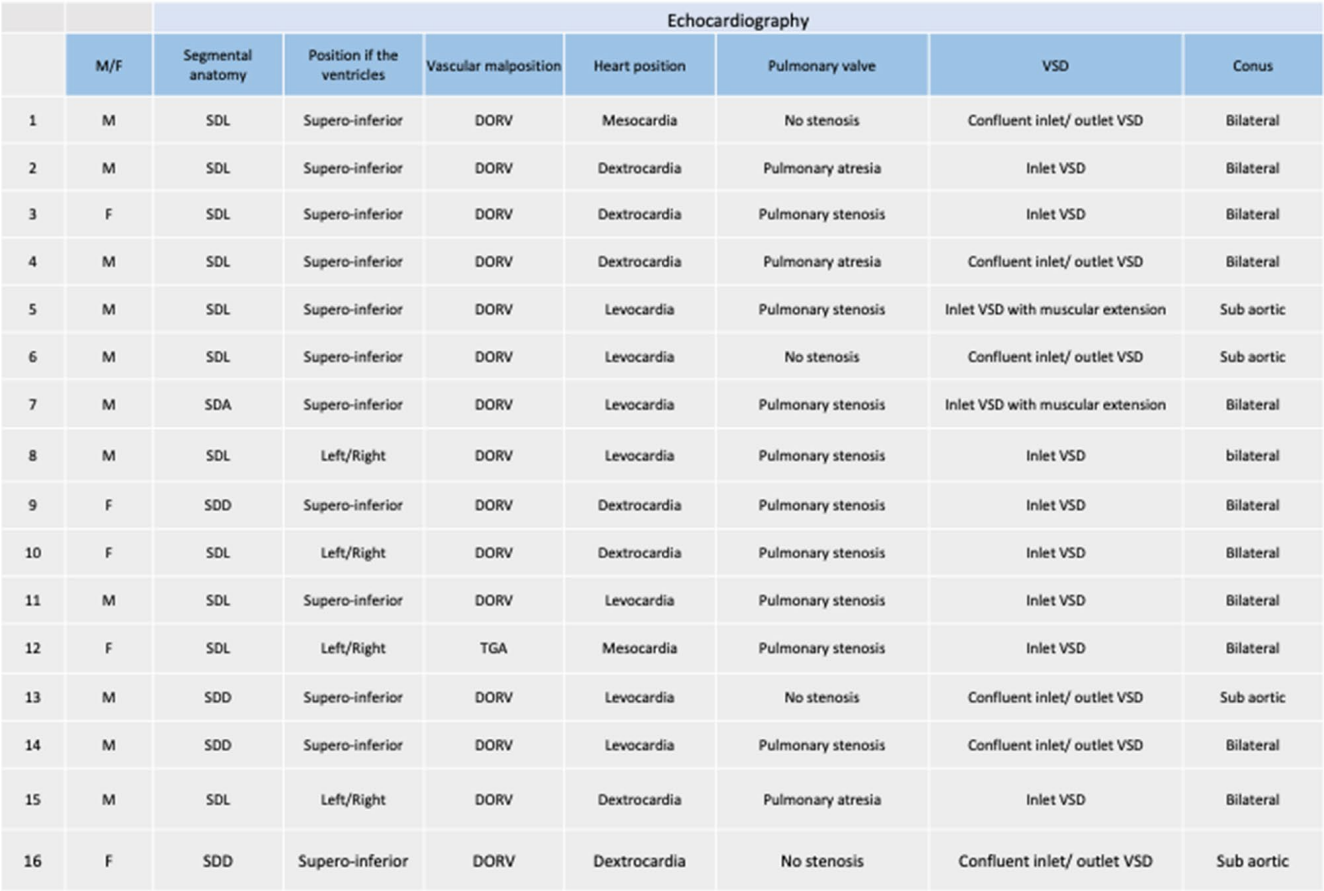
Summary of anatomical characteristics of patients with CCH

### Anatomy

**Fig. 2:**
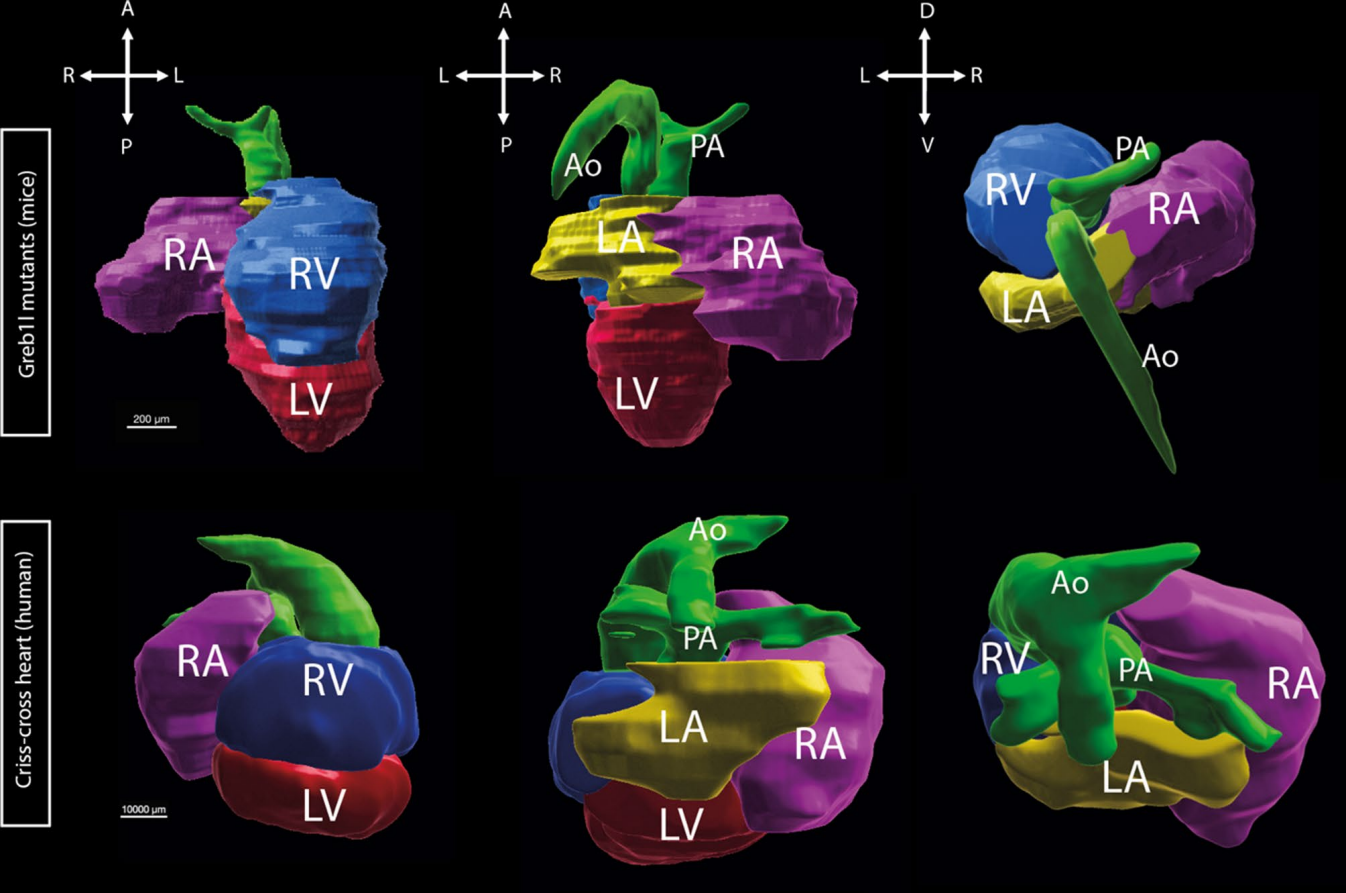
Segmentation of criss-cross heart in the mouse (*Greb1l*^-/-^ mutant) and human. Green: pulmonary artery and aorta; purple: right atriu; Yellow: left atrium; blue:right ventricle; red: left ventricle

Segmental anatomy was SDL in 11 patients (69%), SDD in 4 (25%) and SDA in 1 (6%) (Table 2 and 3). Heart position was levocardia in 8 patients (53%), dextrocardia in 7 (44%) and mesocardia in 1. Twelve patients (75%) had supero-inferior ventricles, which is similar to the mouse model (Fig. 2). Fifteen patients (94%) had double outlet right ventricle with bilateral conus in 11 (75%) and subaortic conus in 4. One patient had transposition of the great arteries. We measured the intrapericardial size of the great arteries by echocardiography in CCH patients compared to controls using the methodology of Omer et al. (Omer et al., 2021) to look for a shortening of the great arteries consistent with a shorter outflow tract. However, our cohort of patients with available neonatal images (*n* = 4) was too small to see any difference.

The pulmonary valve was abnormal (stenosis or atresia) in 12 patients (75%). All patients had a ventricular septal defect opening in the inlet of the right ventricle: inlet defect only in 8 patients (50%), confluent inlet/outlet VSD in 6 (38%) and inlet VSD with muscular extension in 2. Associated cardiac malformations included: cleft mitral valve, straddling tricuspid valve, hypoplastic right ventricle, interruption of the inferior caval vein with azygos return, left superior caval vein.

Fifteen (94%) patients underwent surgery, univentricular repair in 13, biventricular in two. Both patients with biventricular repair had an extension of the VSD in the muscular septum or in the outlet. There was no early nor late death with a median follow-up duration of 10.7 years (0–23).

## Discussion

We report a cohort of 16 consecutive patients with CCH with detailed anatomical description of the cardiac phenotype. The population characteristics align with the existing literature in terms of segmental analysis, mostly SDL (69%) [2, 6]; supero-inferior position of the ventricles (75%) [4, 5]; abnormal ventriculo-arterial connections (100%); and pulmonary stenosis or atresia (80%) [22–24].

The objective of this study was to test the developmental hypothesis that CCH could result primarily from a growth defect of the outflow tract during cardiac development. In *Greb1l* mutant mice, CCH results from an arrested growth of the outflow tract occurring very early, immediately after heart looping. This creates a mechanical constraint triggering an abnormal torsion of the atrioventricular canal and thus repositioning of the ventricles in a supero-inferior configuration [12].

In *Greb1l* mouse mutants the outflow tract stops growing bluntly just after heart looping. At this specific time, the heart tube normally elongates homogeneously, and this homogeneous growth seems to be required to maintain ventricles in a left-right position. Other mouse mutants have shown a shorter outflow tract without supero-inferior ventricles or CCH: *Tbx1* mutants [25], *Pax3* mutants [26] and *Hoxb1* mutants [27]. However, the shortening of the outflow tract in these mutants was less severe or took place later in development. A phenotype similar to *Greb1l* mutants with short outflow tract and supero-inferior ventricular chambers has been observed in a zebrafish mutant for retinoic acid signaling [28].

In our human cohort, all patients with CCH exhibited abnormal ventriculo-arterial connections and an abnormal conus, which is consistent with the hypothesis that a defective growth of the outflow tract is at the origin of the malformation. Indeed, outflow tract defects, including tetralogy of Fallot and double outlet right ventricle, result from a lack of elongation of the outflow tract due to a lack of addition of myocytes from the anterior second heart field, regulated by neural crest cells [29]. The outflow tract is thus too short to achieve complete rotation and exclusive connection of the aorta to the left ventricle, leading to a spectrum of defects, ranging from an overriding aorta with an outlet ventricular septal defect with anteriorly malaligned outlet septum, to tetralogy of Fallot and double outlet right ventricle with subaortic ventricular septal defect [30]. Total absence of rotation of the outflow tract results in double outlet right ventricle with subpulmonary ventricular septal defect and transposition of the great arteries [30]. Recently, Omer et al. [31] showed that newborns with transposition of the great arteries had shorter intrapericardial portions of the great arteries. This finding supports the animal model-based hypothesis that transposition of the great arteries results from a shortening of the outflow tract. In our cohort, we couldn’t find any differences in the size of the great arteries because of the small number of neonatal images available (*n* = 4). However, the constant association of CCH, which is an abnormal atrio-ventricular connection, with an abnormal ventriculo-arterial connection, points to a defective outflow tract development as a possible developmental mechanism of the disease, as demonstrated in *Greb1l* mutant mice.

In our cohort, no candidate variant was identified in *GREB1L* or its TAD. This result is consistent with the observation that *Greb1l* mutant mice exhibit multiple severe malformations and die in utero, suggesting that a complete loss-of-function may be embryonically lethal in humans. Nonetheless, recent evidence indicates that *GREB1L* variants may contribute to complex congenital heart disease, including outflow tract anomalies such as tetralogy of Fallot and double outlet right ventricle, in patients [32]. These findings reinforce the developmental role of *GREB1L* in cardiac morphogenesis. In our cohort, the absence of likely pathogenic or recurrent variants may also reflect an oligogenic or burden-based genetic architecture. However, exploring these hypotheses remains highly challenging in small cohorts, given the rarity of CCH.

All patients also had an inlet VSD. This strongly aligns with an abnormal development of the atrioventricular canal before septation, as reported in the mouse model of CCH [12]. The abnormal development of the atrioventricular valves, perpendicular to each other, does not allow the inlet septum to form correctly. The extension of the VSD in the outlet in 38% of the cases could suggest a secondary role of the anterior second heart field and the neural crest in the outflow tract remodeling [26].

These observations contradict the two main developmental hypotheses previously proposed by morphologists. A repositioning of the ventricles after septation, as proposed by Anderson et al. in 1974, cannot explain the presence of the constant inlet VSD, nor the constant abnormal position of the great vessels. The high frequency of double outlet right ventricle in our cohort indicates that CCH occurs within a developmental window where the outflow tract is still entirely above the embryonic right ventricle, before the transfer of the aorta towards the left ventricle and the achievement of ventricular septation. The second hypothesis of a looping defect [11], is relevant to the observed inlet VSD and abnormal outflow tract. However, only 75% of the ventricles are in a supero-inferior position, and no L-loop was observed in our cohort. Furthermore, abnormal looping in the context of laterality defects such as heterotaxy in mice [33] or human [34] is exceptionally associated with CCH [35]. Indeed, no looping defect was observed in the mouse model of CCH [12], which contradicts Van Praagh’s hypothesis.

The literature about the anatomic type of the VSD associated with CCH is limited, with rare descriptions of perimembranous VSD with inlet/outlet extension [22, 36], muscular VSD and inlet only VSD [5]. Several crucial factors influence the feasibility of biventricular repair in CCH, including the size of the right ventricle, the position of the great vessels, and the associated malformations [37, 38]. Only two patients of the cohort underwent a biventricular type of repair. From a surgical point of view, the constant localization of the VSD in the inlet could account for the difficulties to achieve biventricular repair in CCH. In our study, the two patients who underwent successful biventricular repair had an inlet VSD extending into the muscular septum or the outlet, facilitating the biventricular repair by making easier the connection between the left ventricle and the aorta. Providing a detailed account of VSD localization could enhance surgical decision-making. Advanced techniques such as 3D segmentation and modeling, which can aid in surgical decisions, should consider this aspect.

Due to the rarity of CCH, our cohort is limited to 16 patients. A larger cohort from multiple centers with a detailed anatomic analysis could reinforce understanding of the anatomy, of the developmental origins, and biventricular repair strategies in criss-cross heart.

## Conclusion

CCH in our cohort is always associated with malposition of the great vessels and a VSD, always of the inlet type similar to what is observed in the *Greb1l* criss-cross heart mouse model. This supports the hypothesis of a defective growth of the outflow tract at the origin of this extremely rare malformation, resulting in an incomplete or absent rotation of the great arteries, and abnormal position of the derivatives of the atrioventricular canal. The growth arrest of the outflow tract which occurs very early in development, just after heart looping, explains the specificity of the phenotype compared to other outflow tract malformations, which happen later in development. The inlet VSD is an anatomical cue of an abnormal formation of the inlet septum due to the torsion of the atrioventricular canal, before septation. The VSD location in the inlet further explains the challenges faced by surgeons in repairing this malformation and contributes to the high rate of univentricular repairs in our cohort [14].

## Data Availability

All data generated or analysed during this study are included in this published article.

## Abbreviations

CCH: Criss-cross heart
VSD: Ventricular septal defect
